# SPECT/CT and triple-phase bone scan: A valuable diagnostic approach for identifying indications for secondary patellar resurfacing in patients with unexplained anterior knee pain post-TKA

**DOI:** 10.1186/s42836-025-00300-7

**Published:** 2025-04-02

**Authors:** Chuanlong Wu, Hongyi Wang, Zhijie Chen, Jiong Zhang, Zhihong Liu, Jianmin Feng, Xufeng Jiang, Chuan He

**Affiliations:** 1 Department of Orthopaedics, Shanghai Key Laboratory for Prevention and Treatment of Bone and Joint Diseases, Shanghai Institute of Traumatology and Orthopaedics, Ruijin Hospital, Shanghai Jiao Tong University School of Medicine, Shanghai, 200025 China; 2 Department of Nuclear Medicine, Shanghai Ruijin Hospital, Shanghai Jiao Tong University School of Medicine, Shanghai, 200025 China

**Keywords:** Total knee arthroplasty, Anterior knee pain, Patellar resurfacing, SPECT/CT, Triple-phase bone scan

## Abstract

**Objective:**

The efficacy of secondary patellar resurfacing (SPR) in alleviating anterior knee pain (AKP) following total knee arthroplasty (TKA) remains uncertain. The purpose of this study was to assess the suitability of SPR using single photon emission computed tomography/computed tomography (SPECT/CT) in conjunction with triple-phase bone scan (TPBS).

**Methods:**

We performed a retrospective analysis on a prospectively-enrolled cohort of patients suffering from AKP in the context of TKA. In this cohort, we identified a subset of 17 patients (involving 18 knees), who had unexplained AKP and were potential candidates for secondary patellar resurfacing (SPR). The candidates for SPR were designated the experimental group, and subjects receiving other forms of treatment were referred to as the Control group. The selection for these treatment options was based on the findings from SPECT/CT and triple-phase bone scan (TPBS). Data collection spanned from preoperative to postoperative follow-up periods and encompassed basic demographics, preoperative findings of SPECT/CT plus TPBS, and Knee Society Score (KSS).

**Results:**

SPECT/CT and TPBS revealed focal radionuclide concentration in the patella in 12 patients (13 knees) and in other locations in 5 patients (5 knees) with unexplained AKP, complementing the findings from medical history and physical examinations. The Experimental group showed signs of patellar maltracking or early-stage patellofemoral osteoarthritis (OA) following TKA and received SPR treatment. Postoperatively, the objective knee indicators score was significantly higher than preoperative scores (88.46 ± 5.77 vs. 76.38 ± 7.64, *P* < 0.05). Similarly, the functional activities score was significantly improved postoperatively (74.31 ± 6.68 vs. 50.46 ± 9.01, *P* < 0.05). Patient satisfaction score was substantially elevated after SPR (33.38 ± 2.87 vs. 17.08 ± 5.69, *P* < 0.05). The control group mainly included patients who experienced loosening, periprosthetic joint infection (PJI), or instability. These patients received revision surgeries tailored to their individual pathologies and postoperative follow-ups showed favorable outcomes.

**Conclusions:**

SPECT/CT in combination with TPBS may serve as a valuable tool for assessing the suitability of SPR for the post-TKA management of unexplained AKP.

Video Abstract

**Supplementary Information:**

The online version contains supplementary material available at 10.1186/s42836-025-00300-7.

## Introduction

Total knee arthroplasty (TKA) represents one of the most successful surgical interventions of the twentieth century. It has evolved into a standard treatment for end-stage degenerative knee joint disease, significantly enhancing the quality of life for affected patients. Nonetheless, approximately 20–40% of patients continue to experience postoperative complications such as pain, stiffness, and reduced range of motion. In contrast, only a small fraction, about 4%, of patients report extreme satisfaction, an indicator of a high level of happiness with their surgical outcome [2, 19]. The primary causes of TKA failure encompass aseptic loosening, infection, instability, malposition of the prosthesis, joint fibrosis, patellofemoral joint complications, among others. Among these, pain is a critical determinant impacting the success of TKA. In a seminal study, Mathis et al. examined the characteristics of persistent pain following TKA and found that anterior knee pain (AKP), with pain localized to the anterior aspect of the knee, was present in 89.5% of patients, highlighting its significance in postoperative outcomes [20]. Prior reports indicated that symptoms of AKP following TKA are predominantly characterized by retropatellar or peripatellar discomfort, severely affecting patients’ daily activities [4]. Among the post-TKA complications, patella-related issues (56.7%) and instability (52.6%) emerged as the predominant pathologies, with patellofemoral maltracking being a primary etiology of joint pain. Advances in knee prosthesis design and surgical techniques have led to an incremental reduction in the prevalence of these issues [5, 14]. Nonetheless, research indicated that 5–10% of patients continued to experience AKP after TKA [30].

In general, diagnosis of AKP is fundamentally based on a thorough assessment that covers a detailed medical history taking, physical examination, and diagnostic imaging. Nonetheless, there are instances where routine investigative methods may fail to yield conclusive evidence [30]. In clinical practice, the outcomes of revision surgery are often suboptimal, particularly in patients presenting with unexplained AKP following a normal examination [26]. Previous studies have indicated that the satisfaction rate following secondary patellar resurfacing (SPR) was below 70% [25, 28, 29]. Therefore, it is imperative to first precisely locate the specific lesion site responsible for AKP before proceeding with SPR treatment.

The triple-phase bone scan (TPBS) is renowned for its exceptional sensitivity in detecting bone infections, including osteomyelitis. It is adept at identifying increased osteoblastic activity related to infection, prosthetic loosening, trauma, and neoplasms, enabling earlier diagnosis compared to other imaging modalities such as CT [17]. However, its specificity can be compromised, particularly in cases involving compromised bone [17]. Single photon emission computed tomography/computed tomography (SPECT/CT) offers a significant advantage by providing precise anatomical localization of tracer uptake, which is instrumental in distinguishing between various bone and soft tissue uptakes [10, 12]. This level of precision is not only vital for accurate diagnosis but also critical for effective management planning. The inclusion of delayed-phase SPECT/CT further refines the diagnostic process by reducing the likelihood of false-positive or equivocal results, thereby enhancing the specificity of the diagnosis [17].

The combined use of TPBS and SPECT/CT leads to a more accurate and comprehensive diagnostic approach [6], which is especially valuable in complex cases. This combined strategy may be particularly good at uncovering the underlying causes of patellofemoral issues, such as those arising from malpositioned TKA components or instances of prosthetic loosening [11, 13].

In this retrospective study, we reviewed the medical records of patients presenting with unexplained AKP following TKA. These patients were identified over a decade at our hospital after standard examination procedures, including symptoms, signs, X-ray (involving anteroposterior, lateral and 60° or 90° axial images of the knee), CT, MRI, laboratory tests had excluded infection, loosening, malposition, and other potential causes. Subsequently, we analyzed the outcomes of SPR upon the identification of the underlying cause using SPECT/CT in conjunction with TPBS.

## Materials and methods

### Ethics statement

This study was a retrospective, hospital-based investigation, and was conducted at our institution upon having received approval from the institutional ethics committee.

### Study population

**Fig. 1:**
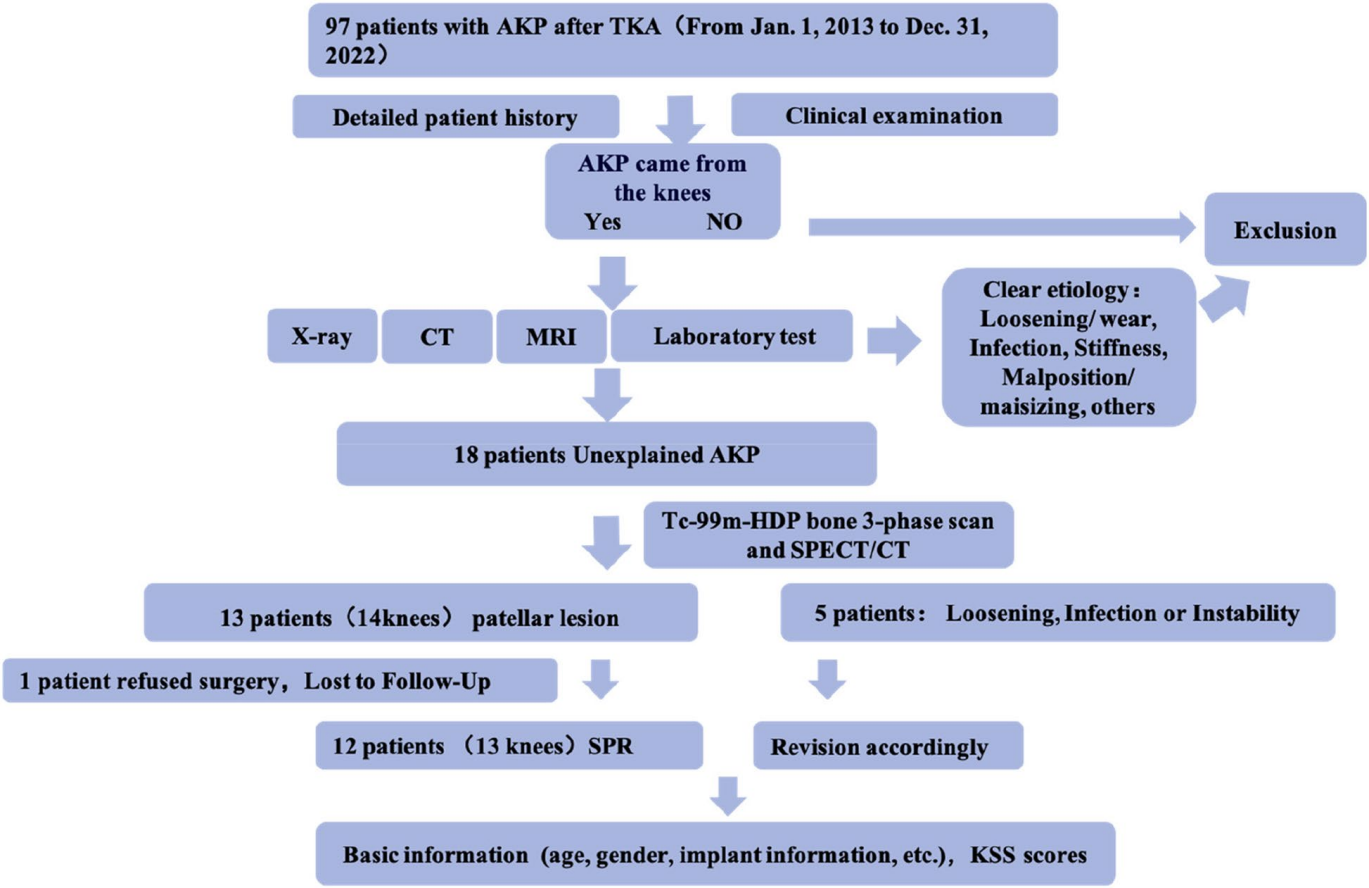
Diagnostic Algorithm for Patients with AKP after TKA. TKA, Total Knee Arthroplasty; AKP, Anterior Knee Pain; SPR, Secondary Patellar Resurfacing

A cohort of 97 patients who developed AKP following TKA was retrospectively evaluated. The patient data spanned from January 1, 2013, to December 31, 2022, and were meticulously reviewed at our hospital. After rigorous exclusion of patients with stiffness, infection, loosening, malposition, patellar maltracking, and other confounding factors through a comprehensive assessment that included evaluation of clinical symptoms, physical signs, radiographic findings (encompassing anteroposterior, lateral, and 60° or 90° axial views of the knee), computed tomography (CT), magnetic resonance imaging (MRI), and laboratory tests, a total of 18 eligible patients were included for further analysis. SPECT/CT in combination with TPBS delineated focal radionuclide uptake on the articular surface of the patella in 13 patients (involving 14 knees). Upon integrating these findings with the patients' medical histories and physical examination results, the criteria for SPR were satisfied. Within this cohort, one patient declined surgical intervention, while 12 patients, involving 13 knees, proceeded to receive SPR. The remaining five knees were subsequently subjected to revision surgeries as indicated (Fig. 1).

### Data collection

We systematically gathered data from clinical records, encompassing operative notes, inpatient charts, and discharge summaries. The collected data included demographic characteristics of the patients, such as age, gender, and body mass index (BMI), initial prosthesis details, preoperative SPECT/CT combined with TPBS findings, and the Knee Society Score (KSS) including the objective knee indicators score (100 points), functional activity score (100 points), patient satisfaction scores (40 points), and patient expectations scores (15 points) at both preoperative and postoperative follow-up intervals.

### Radiology imaging

SPECT/CT imaging was conducted utilizing a state-of-the-art hybrid system, the Symbia T16 (Siemens, Germany), which integrates a pair of low-energy, high-resolution collimators with a dual-head gamma camera and a synchronized 16-slice CT scanner (with a collimation of 16 × 0.75-mm). Patients were administered a standard dose of 740 MBq Tc-99 m methylenediphosphonate (MDP), procured from Shanghai Atomic Science and Technology Pharmaceutical Co. Ltd., China. Planar scintigraphic imaging was executed in triphasic stages: the perfusion phase (immediately post-injection), the soft tissue phase (1 to 5 min post-injection), and the delayed metabolic phase (2 h post-injection). SPECT/CT acquisition parameters included a 128 × 128 matrix size, a 32-degree angular step, and a 30-s frame duration, all performed 2 h post-injection. Data were subsequently processed through an interactive reconstruction on the Syngo work platform (Siemens, Germany), with images rendered in orthogonal axial, coronal, and sagittal planes. The CT protocol adhered to the Imperial Knee Protocol, a low-dose regimen encompassing the hip and ankle joints.

Tracer uptake on SPECT/CT was quantified using a system-based color-coded grading scale ranging from 0 to 10. We meticulously recorded the maximum intensity values for each anatomical region of interest. Subsequently, we derived the ratios of these values to the background tracer activity, which was ascertained from the proximal mid-shaft of the femur. Thereafter, we calculated the mean values along with their standard deviations and conducted a comparative analysis with the contralateral side to discern any significant differences [1–4]. Further, the intraclass correlation coefficient (ICC) analysis was meticulously conducted by a nuclear medicine specialist with a decade of experience and an orthopedic surgeon with a similar tenure. Each expert performed the analysis on three separate occasions, with a week elapsing between each round, in a random sequence to ensure rigor. Throughout this process, both remained unaware of the outcomes from prior assessments.

### Statistical methods

Statistical analysis of preoperative and postoperative KSS was conducted using SPSS version 18.0 software, employing a paired t-test to assess the differences. A *P*-value of less than 0.05 was set as the threshold for statistical significance. The concentration analysis, encompassing the femoral, tibial, and patellar aspects, yielded ICC values exceeding 0.9 when calculated with SPSS, attesting to the high consistency of our findings.

## Results

### Patient characteristics

In this cohort, 12 patients, involving 13 knees, underwent SPR due to patellar lesions identified by the combined use of SPECT/CT and TPBS, complemented by medical history taking and physical examination. The patients aged 70.46 ± 8.12 years on average, ranging from 59 to 84 years. The group consisted of 11 females (12 knees) and 1 male patient. Eleven patients (12 knees) underwent SPR, while one patient received SPR in conjunction with tibial tubercle transposition. The average interval from the primary TKA to SPR was 2.1 years, varying from 0.59 to 6.01 years. The mean follow-up duration after the secondary surgery lasted 6.92 years, spanning from 2.47 to 11.42 years (Table 1).

**Table 1:** Demographics and TKA features of 12 patients (13 knees) receiving secondary patellar resurfacing surgeries

**Case**	**Age**	**Gender**	**Time to SPR (years)**	**Follow-up time (years)**	**Prosthesis**	**SPECT/CT** **Concentration**	**Surgical option**	**Side**	**KSS scores**						
									**Objective knee indicators (100)**		**Functional activities (100)**		**Patient expectations (15)**	**Patient satisfaction (40)**	
									**Pre-surgery**	**Post-surgery**	**Pre-surgery**	**Post-surgery**		**Pre-surgery**	**Post-surgery**
1	84	F	1.18	2.47	Depuy RP	patellar	SPR	Left	80	89	58	74	14	20	32
2	59	F	1.67	2.53	Depuy RP	patellar	SPR + Tibial tubercle osteotomy	Right	69	85	39	78	14	12	34
3	71	F	3.02	2.93	SN legion	patellar	SPR	Right	83	91	48	70	15	20	36
4	66	F	4.85	3.76	Wright aMP	patellar	SPR	Right	73	88	58	76	15	20	32
5	70	F	6.01	5.43	Depuy RP	patellar	SPR	Left	58	74	40	65	14	10	30
6	80	F	1.52	6.51	Wright aMP	patellar	SPR	Right	75	93	44	70	14	14	36
7	79	F	0.72	7.04	Wright aMP	patellar	SPR	Left	75	95	50	71	14	6	36
8	69	F	1.51	8.10	Depuy RP	patellar	SPR	Left	83	89	49	75	15	20	34
9	58	M	1.84	9.22	Depuy RP	patellar	SPR	Right	83	89	50	78	15	20	36
10	71	F	0.86	9.74	Depuy RP	patellar	SPR	Right	85	97	73	92	15	26	34
11	66	F	2.23	10.84	SN legion	patellar	SPR	Right	75	87	47	76	14	20	34
12	64	F	1.27	9.99	Depuy RP	patellar	SPR	Right	71	83	45	67	14	12	26
13	79	F	0.59	11.42	Depuy RP	patellar	SPR	Right	83	90	55	74	14	22	34

*Note*: The term “age” here refers to the time point at which patients underwent SPR surgeries. The average age of the patients was 70.46 ± 8.12 years, with the cohort consisting of 11 females (with 12 knees affected) and 1 male. The average interval from total knee arthroplasty (TKA) to secondary patellar resurfacing (SPR) was 2.1 ± 1.63 years, and the average follow-up duration after SPR lasted 6.92 ± 3.26 years

*Abbreviations*: *TKA* total knee arthroplasty, *SPR* secondary patellar resurfacing, *RP* rotation platform, *aMP *advanced Medial Pivot. All Prostheses were posteriorly stabilized(PS)

Five patients, involving 5 knees, underwent revision surgery due to loosening, periprosthetic joint infection (PJI), or instability, as detected by the combined use of SPECT/CT and TPBS, corroborated by medical history and physical examination. The average age of the patients was 72.8 ± 7.95 years, with a range from 63 to 85 years. The cohort included 3 females and 2 males. Each patient received revision procedures tailored to their specific etiology. The mean interval from the initial TKA to revision surgery was 4.69 years, ranging from 1.44 to 15.26 years. The mean follow-up duration after the revision surgery lasted for 4.47 years, with a variation from 3.38 to 5.3 years (Table 2).

**Table 2:** Demographics and TKA characteristics of 5 patients receiving revision surgeries

**Cases**	**Age**	**Gender**	**Time to revision (years)**	**Follow-up time (years)**	**Prosthesis**	**SPECT/CT** **concentration**	**Diagnosis**	**Surgical option**	**Side**	**KSS scores**						
										**Objective knee indicators (100)**		**Functional activities (100)**		**Patient expectations (15)**	**Patient satisfaction (40)**	
										**Pre-surgery**	**Post-surgery**	**Pre-surgery**	**Post-surgery**		**Pre-surgery**	**Post-surgery**
1	74	Female	15.26	5.30	Zimmer	Tibial	Loosening of tibial prosthesis	Revision of tibial prosthesis and spacer	Right	79	95	46	83	15	12	36
2	63	Female	1.44	4.80	Depuy	Dispersed	PJI	2 stage revision	Left	77	93	42	77	14	12	36
3	85	Female	3.41	4.25	Zimmer	Dispersed	PJI	DAIR	Left	68	88	43	64	14	10	30
4	71	Male	0.80	4.62	Depuy	Dispersed	Instability	Replace spacer	Right	76	91	59	76	15	18	36
5	71	Male	2.57	3.38	Depuy	Dispersed	Loosening	Revision	Left	77	95	41	79	14	12	36

*Note*: The age indicated refers to the time point at which patients underwent revision surgery. The average age of the patients was 72.8 ± 7.95 years, with the group containing 3 females and 2 males. The mean duration from the initial total knee arthroplasty (TKA) to the revision surgery was 4.69 ± 5.99 years, and the average follow-up time post-revision was 4.47 ± 0.72 years

*Abbreviation*: *TKA* total knee arthroplasty, *PJI* Prosthetic Joint Infection, *DAIR* Debridement, Antibiotics, and Implant Retention. All prostheses used were of the posterior stabilized (PS) design

### Following evaluation with SPECT/CT and TPBS, SPR demonstrated favorable outcomes

**Fig. 2:**
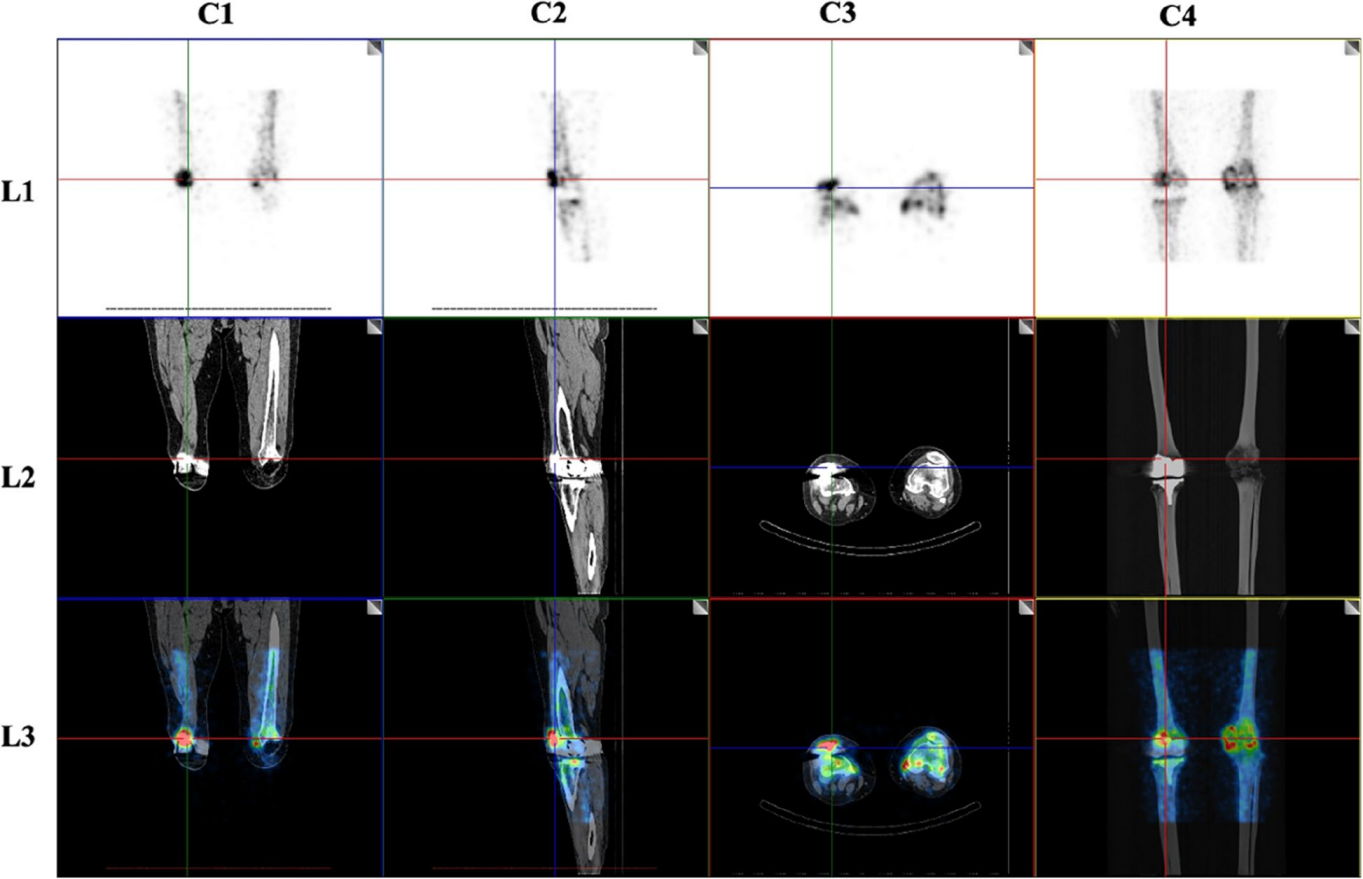
SPECT/CT Imaging of the Knee. The images are presented in various orthogonal planes: coronal (C1), sagittal (C2), and orthogonal axial (C3) planes, alongside a 3D reconstruction (C4). Concurrently, the figure includes images of bone scintigraphy (L1), CT scan (L2), and reconstructed images with a color-coded grading scale (L3)

The objective knee indicators score improved significantly from a preoperative average of 76.38 ± 7.64 to 88.46 ± 5.77 post-SPR at the final follow-up (*P* < 0.05). Similarly, the functional activity score showed a marked enhancement, increasing from 50.46 ± 9.01 preoperatively to 74.31 ± 6.68 post-SPR (*P* < 0.05). Patient expectations were high preoperatively, with a score of 14.38 ± 0.51, and patient satisfaction scores rose significantly from 17.08 ± 5.69 preoperatively to 33.38 ± 2.87 post-SPR at the last follow-up (*P* < 0.05, Table 1). The results suggested that SPECT/CT combined with TPBS offers more nuanced insights into patellofemoral joint degeneration in the diagnosis of unexplained AKP. Images were obtained in coronal, sagittal, and orthogonal axial views, along with 3D reconstructions. Concurrently, bone scintigraphy, CT scans, and reconstructions with a color-coded grading scale were performed (Fig. 2).

### Revision surgery following SPECT/CT and TPBS evaluation yielded satisfactory outcomes

The objective knee indicators score significantly improved from a preoperative average of 75.4 ± 4.28 to 92.4 ± 2.97 postoperatively at the final follow-up (*P* < 0.05). Similarly, the functional activity score demonstrated a substantial increase, rising from 46.2 ± 7.4 preoperatively to 75.8 ± 7.12 postoperatively (*P* < 0.05). Patient expectations were high preoperatively, with a score of 14.4 ± 0.55, and patient satisfaction scores also showed a notable increase from 12.8 ± 3.03 preoperatively to 34.8 ± 2.68 postoperatively at the last follow-up (*P* < 0.05, Table 2). Thus, SPECT/CT in conjunction with TPBS offers more granular diagnostic information for unexplained AKP.

### Cases

#### Case 1

**Fig. 3:**
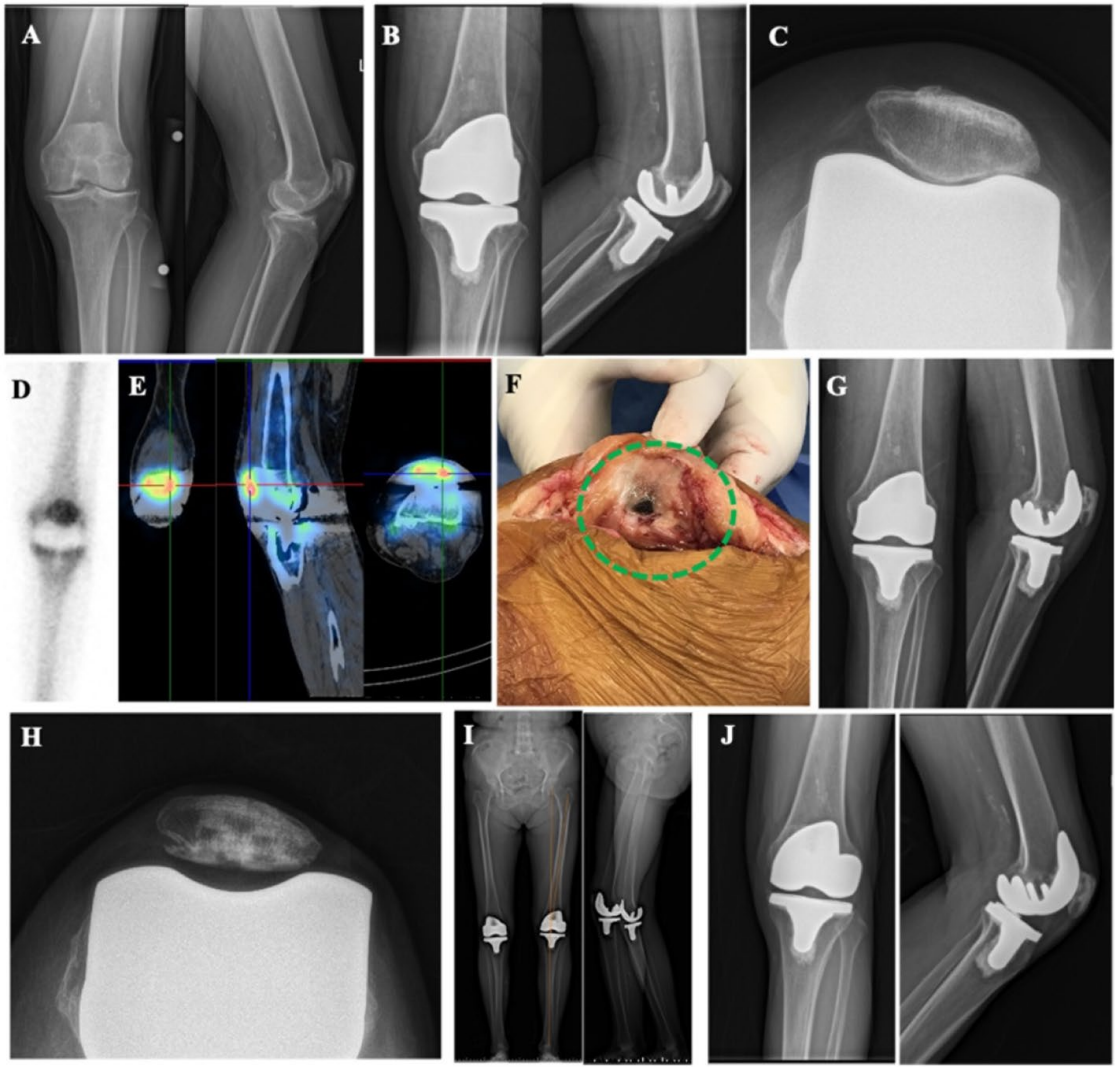
A case of SPR. **A** Standing anteroposterior and lateral radiographs of the left knee reveal narrowing of the medial compartment, characteristic of OA changes. **B** Post-TKA anteroposterior and lateral radiographs of the left knee demonstrate a well-positioned prosthesis with normal alignment. **C** Post-TKA axial radiograph confirms the prosthetic alignment. **D** Triple-phase bone scan highlights radioactive concentration within the left knee joint. **E** SPECT/CT imaging identifies focal radionuclide concentration on the lateral articular surface of the patella. **F** Intraoperative findings include impingement between the lateral articular surface of the left patella and the lateral condyle of the femoral prosthesis, along with cartilage defects and metal debris attachment. **G** Standing anteroposterior and lateral radiographs following SPR. **H** Axial radiograph post-SPR. **I** Full-length radiograph in functional position after SPR. **J** standing anteroposterior and lateral radiographs post-SPR at a two-year follow-up

Figure 3 illustrates a case of SPR. A 79-year-old female underwent left TKA for OA of the left knee (Fig. 3A). She subsequently developed unexplained AKP. Radiographs revealed a satisfactory position and normal alignment of the prosthesis (Fig. 3B and C). TPBS indicated radioactive concentration in the left knee joint (Fig. 3D). SPECT/CT further delineated focal radionuclide uptake on the lateral articular surface of the patella (Fig. 3E). Intraoperative exploration confirmed impingement between the lateral articular surface of the left patella and the lateral condyle of the femoral prosthesis, with cartilage defects and metal debris attachment (Fig. 3F). Pathological examination revealed grayish-yellow, soft tissue sections displaying gray/sallow coloration. The synovial tissue exhibited hyperplasia with chronic inflammation, and local interstitial mucous degeneration was observed. Polymorphonuclear (PMN) cells were fewer than 5 per high power field (HPF). SPR was subsequently performed (Fig. 3G and H), resulting in good function and proper alignment on a full-length radiograph at the functional position (Fig. 3H). The mechanical axis passed through the center of the knee joint, at an angle of 6.3° between the mechanical axis and the femoral shaft axis. At the two-year follow-up, the prosthesis remained well-positioned and functioned effectively (Fig. 3J).

#### Case 2

**Fig. 4:**
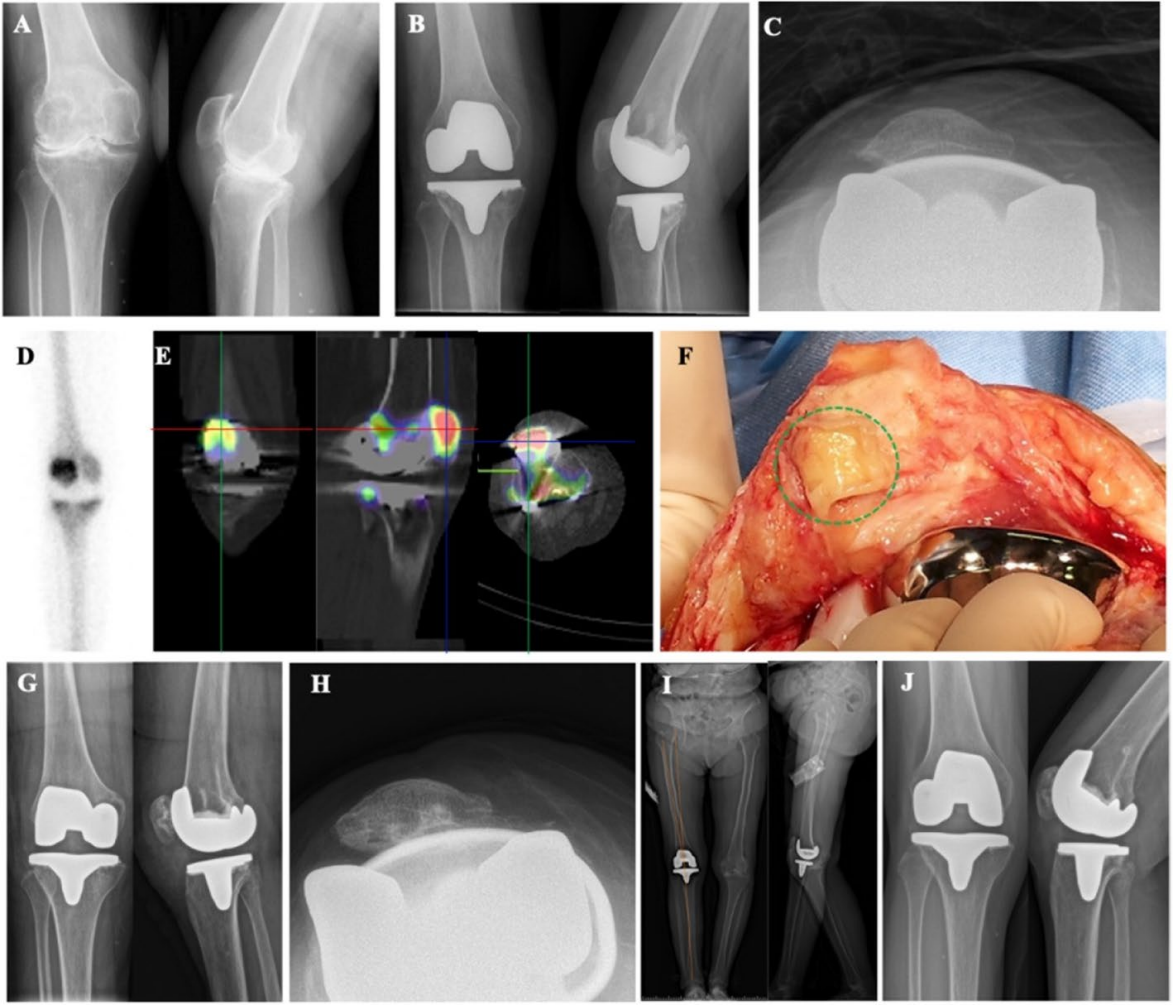
Another instance of SPR. **A** Standing anteroposterior and lateral radiographs of the right knee exhibit narrowing of the medial compartment, indicative of osteoarthritic (OA) changes. **B** Post-TKA anteroposterior and lateral radiographs of the right knee demonstrate a prosthesis that is well-positioned with normal alignment. **C** Axial radiograph post-TKA confirms the alignment of the prosthetic components. **D** Triple-phase bone scan reveals radioactive concentration within the right knee joint. **E** SPECT/CT imaging identifies focal radionuclide concentration on the articular surface of the patella. **F** Intraoperative exploration discloses wear on the articular surface of the right patella accompanied by metal debris. **G** Standing anteroposterior and lateral radiographs following SPR. **H** Axial radiograph post-SPR. **I** Full-length functional radiograph post-SPR. **J** Radiographs at a seven-year follow-up post-SPR

Figure 4 depicts a SPR case. A 78-year-old female underwent right TKA for OA of the right knee (Fig. 4A). She subsequently experienced unexplained AKP. Postoperative radiographs confirmed a satisfactory position and normal alignment of the prosthesis (Fig. 4B and C). TPBS indicated radioactive concentration in the right knee joint (Fig. 4D). SPECT/CT imaging further delineated focal radionuclide concentration on the articular surface of the patella (Fig. 4E). Intraoperative exploration revealed wear on the articular surface of the right patella with the presence of metal debris (Fig. 4F). Pathological examination identified grayish-yellow tissue with chronic synovitis and a PMN count of less than 5 per HPF. Secondary patellar arthroplasty was performed (Fig. 4G and H), resulting in good functional outcomes. A full-length radiograph demonstrated optimal alignment: (1) the mechanical axis passing through the center of the knee joint; (2) an angle of 5.8° between the mechanical axis and the femoral shaft axis (Fig. 4I). At a seven-year postoperative follow-up, the prosthesis remained well-positioned and functioned effectively (Fig. 4J).

#### Case 3

**Fig. 5:**
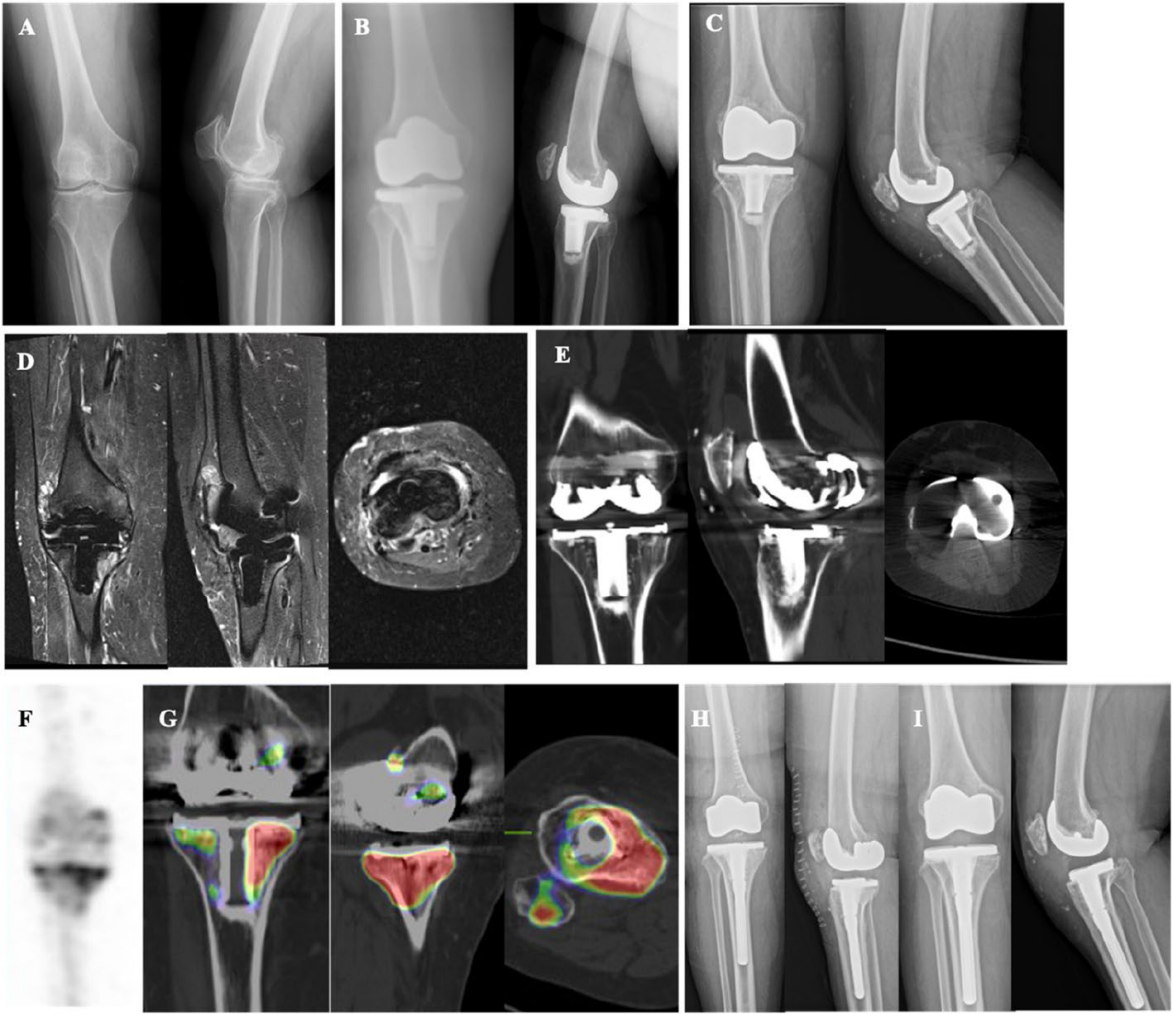
A revision surgery case. **A** Standing anteroposterior and lateral radiographs of the right knee demonstrate narrowing of the joint space, consistent with osteoarthritic (OA) changes. **B** Post-TKA anteroposterior and lateral radiographs of the right knee show a prosthesis in satisfactory position with normal alignment. **C** Anteroposterior and lateral radiographs of the right knee 15 years after the primary TKA. **D** CT scan of the right knee 15 years post-TKA. **E** MRI of the right knee 15 years post-TKA. **F** Triple-phase bone scan reveals radioactive concentration in the right knee joint. **G** SPECT/CT imaging identifies focal radionuclide concentration in the lateral tibia. **H** Intraoperative exploration discloses impingement between the lateral articular surface of the left patella and the lateral condyle of the femoral prosthesis, with cartilage defects and metal debris attachment. **I** Standing anteroposterior and lateral radiographs following revision surgery. **J** Standing anteroposterior and lateral radiographs at a one-year follow-up post-revision

Figure 5 illustrates a revision surgery case. A 59-year-old female who had undergone right TKA for knee osteoarthritis (OA) presented with unexplained AKP 15 years post-TKA (Fig. 5A and B). Radiographs, CT, and MRI scans suggested the possibility of prosthetic loosening or PJI (Fig. 5C, D, and E). TPBS indicated radioactive concentration in the right knee joint (Fig. 5F). SPECT/CT imaging further delineated focal radionuclide concentration in the lateral tibia (Fig. 5G). Intraoperative exploration confirmed the loosening of the tibial prosthesis. Pathological examination revealed grayish, soft tissue with degenerative changes in the synovial tissue and a PMN count of less than 5 per HPF. Revision surgery of the tibial prosthesis was performed (Fig. 5H), resulting in good functional outcomes. At a one-year follow-up, the prosthesis remained well-positioned and functioned effectively (Fig. 5I).

## Discussion

While TKA has achieved considerable success in addressing end-stage knee OA and severe deformities, the diagnosis and management of postoperative unexplained AKP still pose significant challenges. In this retrospective study, we reviewed the data of patients with unexplained AKP at our hospital over a period of ten years. The focal patellar lesions identified by SPECT/CT showed that the primary etiologies were patellar maltracking and early-stage patellofemoral OA. Encouragingly, all patients treated with SPR experienced favorable outcomes.

A comprehensive diagnostic protocol for patients presenting with AKP following TKA has been established, as outlined in Fig. 1. This protocol commenced with a thorough medical history taking and physical examination to assess potential instability and the alignment and rotational positioning of the prosthesis using X-ray and CT imaging. MRI, in conjunction with laboratory tests, was employed to detect possible intra-articular inflammation. TPBS was frequently utilized to identify occult infections associated with knee prostheses and to assess for prosthetic loosening. Typically, common causes such as malposition of tibial and femoral components, infection, loosening, and patellar maltracking can be identified through these methods. However, TPBS often falls short in pinpointing the cause of AKP when local changes in the patellofemoral joint are subtle.

It remains controversial whether patella resurfacing should be performed during primary TKA [7, 15, 18, 27, 32]. Patellar resurfacing in primary TKA addresses issues such as suboptimal patellar tracking and audible clicks during trial testing, and is particularly beneficial for knees with a hypoplastic lateral femoral condyle. However, this procedure presents additional challenges, potentially carries the risk for complications, and may not be feasible in cases where the patella is excessively thin, precluding effective resurfacing [7, 15, 18, 27, 32]. Deroche et al. conducted a prospective randomized controlled trial involving 245 consecutive patients (250 knees) who underwent TKA. Their study revealed no significant benefits of patellar resurfacing in terms of mid-term clinical and radiological outcomes [8]. Parvizi et al. followed up 39 patients (41 knees) who underwent SPR for AKP and found that eight patients (eight knees) reported dissatisfaction with the surgical outcomes. Nevertheless, the clinical and functional knee scores were significantly improved across the entire cohort [25]. Thomas et al. reported that only 44% of patients felt that SPR effectively alleviated their AKP [28]. A meta-analysis revealed that 64% of patients expressed satisfaction with SPR [29]. The observed low satisfaction rate implies that a thorough preoperative assessment is essential to the optimization of outcomes in AKP-associated SPR. SPECT/CT examination may offer more granular local information, which is crucial for diagnosis. Murer et al. found that the sensitivity and specificity of SPECT/CT in detecting patellofemoral OA were 96.5% and 96.2%, respectively [24]. The sensitivity and specificity for detecting loosening of the tibial prosthesis were 96.0% and 100%, respectively. Similarly, the sensitivity and specificity for detecting loosening of the femoral prosthesis were 95.0% and 100%, respectively [24]. Another study identified a significant correlation between pain and patient characteristics on the basis of findings from SPECT/CT [21]. In this study, SPECT/CT precisely identified the site of poor trajectory and patellar impact in light of localized abnormal nuclide concentration in the patella. This result was corroborated by intraoperative findings, which revealed that the patellar defect was situated on the exterior and inferior articular surface. Consequently, 12 knees underwent SPR. In one case, an abnormal femoral condyle rotation, attributed to the malunion of an old femoral fracture, was identified. This knee was concomitantly treated with SPR and tibial tubercle transposition adjustment, leading to a significant enhancement in the patient's function and satisfaction postoperatively. However, one patient continued to experience dull pain and discomfort in the knee joint after surgery, which aligns with literature suggesting that SPR may not fully alleviate symptoms in some patients. Nevertheless, the patient's satisfaction was markedly improved following SPR.

Ultimately, axial imaging of the patella plays a crucial role in the diagnosis of patellofemoral joint pathologies, including OA and maltracking. Merchant et al. introduced a traditional skyline-view technique for acquiring axial patellar images, advocating that the knee should be flexed to approximately 45° for optimal visualization [23], whereas Laurin et al. posited that if the knee is flexed at an angle exceeding 20°, there is a risk of overlooking patellofemoral pathology due to the contraction of the quadriceps muscle [16]. Ficat et al. [9] and Peter et al. [31] championed assessment of a sequence of skyline radiographs at specific angles of knee flexion—30°, 60°, 90° for one series, and 30°, 45°, 60° for another. Conversely, Bhattacharya et al. did not support the routine use of the skyline view in radiological assessments [1, 3], and McDonnell et al. proposed that skyline patellofemoral radiographs are capable of ruling out only the advanced stages of degenerative changes [22]. Furthermore, the patellofemoral joint space is often not conspicuous in skyline images due to the proximity of the knee to the radiography cassette. In this study, we included axial images of the patella, taken at 60° or 90° of knee flexion, as part of our standard examination protocol. AKP was primarily induced during physical examination when the knee was extended during the final 15°–30° of extension from flexion, a motion commonly observed when donning pants, colloquially referred to as the “Wear Pants Sign”. As a result, we hypothesize that the impact between the patella and the femoral prosthesis occurs during the transition through 15°–30° of flexion.

This study has several strengths. Firstly, to the best of the authors' knowledge, it is the first systematic investigation to assess the efficacy of SPR using SPECT/CT in the context of AKP following TKA; secondly, it covered an extended follow-up duration, with an average of 6.92 years and a maximum of 11.42 years. Nonetheless, the study is not without limitations. Firstly, it was of retrospective design. Secondly, it was a single-center study with a limited sample size, and thirdly, it involved more irradiation exposure.

In conclusion, the use of SPECT/CT in conjunction with TPBS may be an appropriate approach to determine if SPR is a viable treatment option for AKP associated with TKA.

## Data Availability

We have included our raw data in a supplementary file. All data and materials are true and transparent. This manuscript has not been published elsewhere and is not under consideration for publication by another journal.
